# Epigenetic Regulation of Innate Immunity by microRNAs

**DOI:** 10.3390/antib5020008

**Published:** 2016-04-01

**Authors:** Chandra S. Boosani, Devendra K. Agrawal

**Affiliations:** Department of Clinical & Translational Science, Creighton University School of Medicine, Omaha, NE 68178, USA; chandraboosani@creighton.edu

**Keywords:** microRNAs, epigenetic gene regulation, innate immunity, TLR signaling, suppressor of cytokine signaling (SOCS), interleukin inducible pathways, NF-kB signaling, RIG-1 and IPS-1 mediated signaling, macrophage polarization, regulation of apoptosis

## Abstract

The innate immune response, which is usually referred to as the first line of defense, protects the hosts against pathogenic micro-organisms. Some of the biomolecules released from the pathogens, such as proteins, lipoproteins and nucleic acids, which are collectively termed as pathogen-associated molecular patterns (PAMPs), elicit signaling mechanisms that trigger immune responses in the hosts. Pathogen recognition receptors (PRRs) on the host cells recognize these PAMPs and initiate intracellular signaling through toll-like receptors (TLRs), RIG-I-like receptors (RLRs), and other pathways which induce production of pro-inflammatory cytokines and type I interferons. Recently, different members of tripartite motif containing proteins (TRIM) family of proteins were identified to intercept and regulate these cellular pathways. Specific targets of TRIM proteins have been identified and their molecular mechanisms were unraveled and identified unique domains involved in protein-protein interactions. Though innate immunity represents a tight and well conserved immune system in the host, gene expression in innate immunity was identified to be influenced by several epigenetic mechanisms including regulation by microRNAs (miRNAs). In this review, we present critical analysis of the findings on the identification of specific miRNAs that modulate expression of target genes involved in the regulation of innate immunity.

## 1. Introduction

The dynamic nature of pathogens, and their ability to invoke modified strategies to escape host immunological recognition, projects challenges in the host to combat invading pathogens. One of the initial and essential parts of the host immune system in waging defense against the pathogen is to initiate innate immune response. This first line of defense will also induce adaptive immunity at a later stage towards a continuous defensive effort against the pathogens. Execution of the innate immune response is often initiated through Toll-like receptors (TLR) signaling pathways. Typically, ligands from the pathogens are recognized by TLRs that are present on the extracellular transmembrane and also on the surface of endosomes. TLRs then initiate intracellular defense response against the invading pathogens. Though immune response pathways are highly conserved, the response for the same pathogen varies between individuals. This raises the question: is there an epigenetic mechanism that contributes to these variations? It is now profoundly established that epigenetics play a major role in gene regulation. Besides the role of histones in epigenetic genome modifications to alter gene expression patterns, epigenetic gene regulation by miRNAs is widely studied in the recent past which effectively controls gene expression. The mechanism of non-heritable gene regulation might address the differences in immune tolerance even between the homozygotic twins. With the advent of miRNA arrays, evidence is accumulating to support definitive role of specific miRNAs in regulating innate immunity.

A clear role of miRNAs in regulating TLR-induced intracellular signaling in innate immunity was previously reported wherein, THP-1 monocytes activated with lipopolysaccharide (LPS) were found to initiate the TLR4 signaling and contribute to the induction of the microRNA miR-146 [1]. Biogenesis of miRNAs is the result of endonuclease activity of two major ribonucleases, Dicer and Drosha. An apparent role of Dicer in miRNA-mediated gene regulation became evident from the gene ablation studies of both Dicer alleles in mice where, absence of Dicer alleles proved to be embryonically lethal and embryos died around day 8 of post coitum [2]. It is clear that Dicer besides processing double stranded RNA molecules also processes small RNA molecules and generates microRNAs [3,4,5]. Initial evidence on the role of Dicer in the maturation of miRNA came from *in vitro* studies in HeLa cells. Upon transfection of these cells with Dicer-specific siRNA, increased accumulation of let-7 precursor products were observed [6]. The RNA binding protein Lin28, which was identified as an inhibitor of pri-miRNA processing, binds to different members of let-7 precursor miRNAs and prevents processing mediated by Drosha. Since Lin28 is selectively expressed in embryonic cells, cellular and developmental specific functions can be attributed to Drosha and miRNA processing [7]. Characteristically, miRNAs have been identified to regulate post-transcriptional gene expression of the target genes. Besides this, expression of miRNAs itself was identified to be post-transcriptionally regulated and their differential processing into mature miRNAs from the precursors could contribute to their temporal and spatial expression [8]. Recently, miRNAs were also identified to regulate different intracellular signaling mechanisms in the innate immune cells. In PAMP-challenged monocytes, miRNAs, such as miR-9, miR-21, miR-132, miR-146, and miR-155, have been reported to regulate cellular homeostasis [1,9,10,11,12,13,14,15].

Phylogenetic analysis revealed that Dicer shares high homology with DExH/D and Helicase C conserved domains of retinoic acid-inducible gene-1 (RIG-I), melanoma differentiation-associated protein 5 (MDA5), Laboratory of genetics and physiology (LGP2) and eIF4A. Existence of such a similarity among these proteins suggests that they may have a common regulatory role, and it has been shown that Dicer can potentially interact with RIG-I like receptors [16,17]. These reports unequivocally establish a clear role of Dicer and its chief functions in processing and generating miRNAs regulating innate immunity. From this perspective, in the following section we provide published evidence on the identified miRNAs and their characteristic role as epigenetic gene regulators in innate immunity.

## 2. MicroRNAs Regulate Innate Immune Response

**Figure 1:**
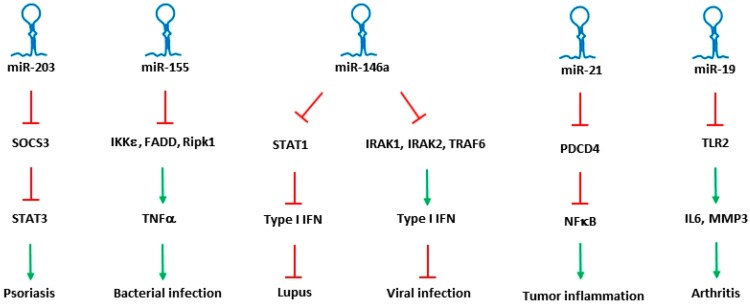
Key miRNAs involved in the regulation of innate immune response. miRNAs along with their specific targets and downstream mediators associated with different innate immune disorders are shown. Inhibition by miRNAs was shown as ⊥ highlighted in red, while their inducing effect is shown as ↓ highlighted in green.

A coordinated role of both adaptive and innate immune signaling is required to protect the host from a variety of pathogens. During the entry of bacteria or the virus particles, instant recognition of the pathogens is mediated through adaptive immune response. Subsequent endocytosis of the pathogen triggers innate immune responses, which are critically required to initiate a strong defense. Once a defense mechanism is fully activated, innate immune pathways in turn help in inducing adaptive immune system. In regulating the immune responses, microRNAs specific to and expressing in different immune cells were identified, and their role in differentiation, maturation, proliferation and activation of immune cells were determined. Though the bulk of literature shows role of microRNAs in both adaptive and innate immune responses, only microRNAs that were identified with a clear role in regulating innate immune responses are described here. List of miRNAs that are involved in the regulation of innate immune pathways are shown in Table 1, and a few of the miRNAs that were identified to regulate different innate immune diseases are shown in Figure 1.

### 2.1. MicroRNAs Inhibit SOCS Proteins in Innate Immunity

Mechanisms underlying the role of suppressor of cytokine signaling protein 1 (SOCS1) in innate immunity appears to be through inhibition of interleukin-1 receptor-associated kinase IRAK1. As a result, TLR-mediated NF-κB signaling is affected which is downstream to IRAK1. Mice lacking SOCS1 were found to be highly susceptible to LPS-triggered septic shock and were also pathologically sensitive. Notably, SOCS1-deficient mice showed increased type I and type II Interferon (IFN) signaling, and mice died at three weeks of age [18,19,20,21]. The mechanism of SOCS1 by which it inhibits the effects of IFNα was reported to be mediated through its interaction with the catalytic domain of Tyk2. Phosphorylation of Tyr-1054 and Tyr-1055 within this catalytic domain was shown to be crucial for its interaction with SOCS1 protein [22]. HeLa cells when infected with vesicular stomatitis virus (VSV), displayed inhibition of the antiviral response of IFNα and IFNγ only in cells that expressed SOCS1 and SOCS3 but not SOCS2 [23]. Recently, we reviewed regulation of SOCS3 expression and factors that contribute to its epigenetic modifications, and summarized how the functions of SOCS3 are controlled by different interleukins in waging an immune response [24]. During infection, HSV and Epstein-Barr virus (EBV) were found to stimulate expression of SOCS3 to suppress the production of type I IFN and attenuate the downstream signaling events and cellular responses [25,26]. The above studies establish a clear role of SOCS proteins in regulating innate immunity and highlight recent developments on the specific role of different microRNAs regulating expression of SOCS proteins in innate immunity.

Further, in mouse peritoneal macrophages infected with VSV, expression of miR-155 was induced through RIG-1-dependent pathway, which subsequently inhibited SOCS1 expression. This resulted in enhanced expression of type I IFN and its antiviral response in inhibiting viral replication in the host cell. The VSV-induced expression of miR-155 contributes to the positive feedback regulation of type I IFN by targeting SOCS1 expression [14]. In cultured dendritic cells (DCs) from miR-155 null mice, reduced levels of IL-12p70 were observed which was essential for NK cell activation [27]. Further, silencing of SOCS1 in DCs was also shown to enhance production of interleukin-12 (IL-12), which supports the role of miR-155 as a SOCS1 modulator through positive feedback regulation [28].

In Huh7 human hepatoma cells, silencing of miR-122 was not only reported to enhance IFNα signaling and reduce SOCS3 expression by inducing promoter methylation, but it also enhanced STAT3 activation [29]. IFN-1 treatment is the current standard of therapy to treat HCV infection. Treatment with IFN-l increases the expression of SOCS3 and the microRNA miR-122 which may have contributed to the inhibition of type I IFN signaling possibly by inducing the gene expression driven by interferon-stimulated response elements (ISRE) [29]. In psoriatic plaques, characteristic up-regulation of miR-203 classified it as a psoriasis specific signature miRNA. SOCS3, which negatively regulates IL-6 and IFNγ signaling, is a direct target of miR-203. Accordingly, up-regulation of miR-203 in psoriasis was found to inhibit SOCS3 expression leading to sustained activation of STAT3. As a result, the inflammatory stimulus in the keratinocytes of skin were endured which might have elevated this pathology [30].

### 2.2. Role of miR-223 in Macrophage Polarization

Both in human and mice, expression of the microRNA, miR-223, unequivocally confines to the hematopoietic cells. With an established role in innate immunity, miR-223 was identified to target Mef2c transcription factor, which is essential for the proliferation of myeloid progenitor cells, and the differentiation and activation of granulocytes [31]. In acute promyelocytic leukemia (APL), myeloid cell differentiation was induced by retinoic acid treatment, both *in vitro* and *in vivo* [32,33]. However, whether the treatment with retinoic acid initiates any intra-cellular signaling through Retinoic acid inducible gene 1 (RIG-1) was not clear. Treatment with retinoic acid increased the expression of the transcription factor CEBPα that prevents NFI-A function to repress miR-223. Therefore, induction of CEPBα was identified as a mechanism to promote the expression of miR-223 [32]. CEBP transcription factors were also identified to play a critical role in the macrophage development, and activation of CEBPβ is required for the polarization of M1 macrophages induced by TLR ligands. However, a definitive role of CEBPα in macrophage polarization is yet to unraveled. Recent evidence shows that treatment with IL-4 induced activation of M2 macrophages leads to a dramatic increase in the expression of miR-223 in mouse bone marrow derived macrophages. Elevated levels of pro-inflammatory cytokines, such as Tumor Necrotic factor α (TNFα) and IL-6, and decrease in M2 associated markers, PPARγ and Arginase-1, were also reported in miR-233 null mice [34]. In addition, miR-223 down-regulates the expression of lymphoid transcription factor Lef1, which delays macrophage trans-differentiation [35]. These observations support a definitive role of the microRNA miR-223 in macrophage polarization.

### 2.3. miR-29 Regulates Apoptosis during Innate Immunity

Three mature microRNAs, miR-29a, miR-29b and miR-29c, share a common seed region and have been classified as miR-29 family members. Although their distribution, location of actions and gene specificities are different, they all have been identified to be involved in the pro-apoptotic functions [36]. The Natural Killer (NK) cells from mice infected with Listeria monocytogenes exhibit increased expression of IFNγ with concomitant decrease in the expression levels of miR-29a and miR-29b. Similar observations were reported in NK cells that were stimulated with Poly IC or PMA, where increased transcripts of IFNγ and lower expression of miR-29a were reported [37]. Human pulmonary epithelial cells when infected with either of the subtypes of Influenza A virus, H1N1 or H3N2, were found to elevate the expression levels of the microRNA miR-29c. The expression of the anti-apoptotic gene BcL2L2, which is an effective target of miR-29c, was also inhibited in the transfected cells that overexpressed miR-29c, indicating a clear role of miR-29c in regulating the apoptotic pathway during activation of innate immunity [38]. In addition, in Hepatocellular carcinoma cells (HCC) that were infected with hepatitis B virus (HBV), overexpression of miR-29c effectively suppressed the expression of TNFα-induced protein 3 (TNFAIP3) which plays an essential role in regulating inflammation and immunity [39]. In addition, the microRNA miR-29b was reported to be down-regulated in cholangiocarcinoma cells expressing high levels of the anti-apoptotic protein McL-1 which is a member of BcL2 family. The study also showed induction of tumor necrosis factor-related apoptosis-inducing ligand (TRAIL), which sensitized the tumor cells to cytotoxic agents when miR-29b was overexpressed [40]. These reports provide ample evidence demonstrating a prominent role for miR-29 family of microRNAs in regulating cellular apoptosis.

### 2.4. Role of miR-155 in Innate Immunity

The miR-155 has been identified as an important regulator of many hematological disorders with an essential role in different pathways associated with host immunity. Intraperitoneal injection of sub-lethal doses of LPS in mice has been shown to induce the expression of miR-155 in bone marrow cells, and this increase in miR-155 was correlated with the expansion of granulocytes/monocytes [41]. Since expression of miR-155 was induced in macrophages and dendritic cells through TLR-3 and TLR-4 signaling pathways, it suggests that miR-155 has a prominent role in regulating innate immunity [12,42]. In human mesangial cells, treatment with both TNFα and IFNγ were shown to impart a synergistic effect in the induction of miR-155 expression. While this induction is mediated through TAB-2/NF-κB pathway, the induced miR-155 was found to negatively regulate expression of TAB-2 and IFNγ-inducible protein-10, which suggests the existence of a negative feedback regulation during inflammation [43]. In mice with LPS-induced inflammation, silencing of miR-155 resulted in restored expression of CEBPβ transcription factor and inhibition of granulocyte colony-stimulating factor (G-CSF) [44]. SHIP1, which is an important regulator of immune functions, inhibits the PI3K/Akt signaling pathway, and in miR-155 null mice, macrophages showed repressed SHIP1 activity [13]. HEK-293 cells when transfected with miR-155 showed enhanced expression of TNFα, and the same was observed in miR-155 transgenic mice that were challenged with LPS. Therefore, it was presumed that miR-155 might aid in stabilizing the mRNA transcripts of TNFα [45]. The target transcripts of miR-155 include inducible IκB kinase (IKKε), FADD and Ripk1. These proteins aid in the activation of LPS-induced TNFα effector pathways. This anomaly clearly indicates that either a negative feedback regulation exists or the delayed onset of these targets may have a regulatory role in the expression of miR-155 [45].

### 2.5. microRNA miR-146, a Key Player in Innate Immunity

Evidence from the recent literature suggests a very prominent role of the microRNA miR-146a in many human diseases such as arthritis, psoriasis, COPD, diabetes, cancer, bacterial and viral infections, and in many inflammation associated disorders. In murine peritoneal macrophages, vesicular stomatitis virus (VSV) infection upregulates the expression of miR-146a. The induced miRNA was shown to negatively regulate type I IFN by inhibiting its expression. This inhibition of type I IFN by miR-146a, in turn, promotes VSV replication and contributes to immune evasion. The same study also showed that miR-146a selectively targets IRAK1, IRAK2 and TNFR-associated factor 6 (TRAF6) and inhibits their expression, and this inhibition leads to suppression of RIG-1-mediated production of type I IFN [46]. In human monocytes, LPS-induced production of miR-146a and miR-146b was mediated through NF-κB pathway, indicating a negative feedback regulation between TLR pathway and cytokine receptor signaling [1]. The latent membrane protein-1 (LMP1) encoded by Epstein-Barr virus (EBV), which was identified as an onco-protein, was also able to induce the expression of miR-146a through NF-κB dependent pathway [47,48]. Increased expression of miR-146a was also observed in Burkitt’s Lymphoma cells that were infected with EBV which suggests that additional viral proteins or viral miRNAs or virus induced miRNAs may contribute to the induced expression of miR-146a [49]. In patients with chronic to severe Hepatitis B infection, lower levels of plasma complement factors C3 and C4 were reported [50]. In a recent study, the HBV X protein from Hepatitis B virus was found to promote expression of the microRNA miR-146a, and its increase was correlated with the down-regulation of Complement factor H (CFH). Thus, CFH was identified as a direct target of miR-146a, and down-regulation of CFH was found to induce inflammation in the liver [51]. Negative regulation of type I IFN through TLR-7 pathway by miR-146a was identified to inhibit the expression of STAT-1 and interferon regulatory factor 5 (IRF-5) in systemic lupus erythematosus (SLE) patients [52]. The same study further confirmed that in *in vitro*, over expression of miR-146a reduced the expression of STAT-1 which correlates with the earlier reports from lupus patients and in a mouse model where expression and activation of STAT-1 was reported [53,54]. IL-1β has been shown to induce several pro-inflammatory molecules such as G-CSF, HMGB1, IFN-γ, IL-6, IL-8, IP-10, MCP1, MIP-1β, and TNFα. During severe inflammation, expression of IL-1β was correlated with upregulation of miR-146a with concomitant down regulation of IL-8 and RANTES. However, miR-146a itself could not affect the signaling pathway of IL-1β or the mRNA levels of IL-8 and RANTES which indicates the existence of negative feedback regulation in the presence of high concentrations of IL-1β [55]. Interestingly, IL-1β did not alter the expression of miR-146a in human small airway epithelial cells, but treatment with TNFα or the mechanical stimulus induced by oscillatory pressure was found to induce about two-fold increase in the expression of miR-146a [56]. IL-6 and COX-2 are two exceptionally important pro-inflammatory markers which initiate inflammatory response in most pathological infections. In human astrocytes, it has been reported that transfection with the microRNA miR-146a reduced the expression levels of IL-6 and COX-2 mRNAs in IL-1β-stimulated cells [57].

## 3. Viral microRNAs that Regulate Innate Immunity

Identification and cloning of viral miRNAs was initially reported in Burkitt’s lymphoma cells that were infected with EBV [58]. The increasing list of virus encoded miRNAs suggests their ability to regulate different functional aspects of host cellular machinery. It is logical to anticipate viral miRNAs to have the potential to target and manipulate the host gene expression for their advantage to grow and multiply, and may also suppress the host immune responses against the invading virus. The Kaposi’s sarcoma-associated herpesvirus (KSHV) is among the well-studied viruses that have been identified to encode viral miRNAs. To date, 12 kshv-miRs have been identified as per the miRBase database. A current list of about 502 mature viral miRNAs that were listed at the miRBase database is shown in Table 2 [59,60,61,62,63]. In addition, a detailed and increasing list of viral miRNAs and their targets can be found in the VIRmiRNA database [64].

In an interesting approach, 293 cells were transfected with the miRNA gene cluster amplified from the KSHV genome which harbored ten of its miRNAs. Gene expression analysis using microarray reportedly showed significant changes in the expression profiles of 81 genes. Specifically, thrombospondin expression was downregulated indicating it as a target of the KSHV cluster of miRNAs. Since thrombospondin-1 is known to activate TGFβ, down-regulation of thrombospondin-1 by this miRNA cluster was correlated with the decreased expression of TGFβ, which presumably affected the TGFβ-mediated signaling [65]. The molecular mechanism of the inhibition of TGFβ by KSHV microRNA miR-K12-11 was recently identified to be mediated through SMAD5 which is also a direct target of miR-K12-11 [66]. In a recently reviewed article, to understand the miRNA targetome of KSHV, 13 unique target genes have been identified which included NF-κB/IKK signaling molecules. These findings undoubtedly show the regulatory roles of KSHV miRNAs in modulating innate immune signaling in the host [67].

## 4. RIG-1 and MDA-5 Signaling in Innate Immunity

During pathogen recognition in the host, the pattern recognition receptors (PRRs) on the immune cells recognize specific pathogen signatures which are collectively termed as pathogen-associated molecular patterns (PAMPs). As a result, robust signaling events are initiated which would lead to the production of pro-inflammatory cytokines, type I IFN and subsequent cellular signaling induced by type I IFN. On the other hand, viruses for their parasitic nature depend on the host cellular and biochemical system to replicate and propagate. Detection for the presence of foreign nucleic acids inside the cells is recognized by host PRRs which initiates the defense response. However, it is not still completely clear as to how the host cells are able to differentiate the self and non-self nucleic acids.

**Figure 2:**
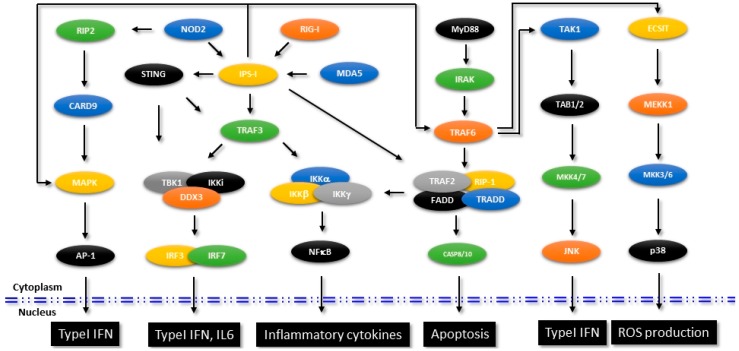
Signaling mechanisms in innate immunity. Figure shows the identified signaling mechanisms that are involved in the regulation of innate immune pathways. Specific molecules that intersect other pathways affecting intracellular functions are shown.

Detection of the viral nucleic acids in the host cells and induction of antiviral innate immune response were identified involving a well-coordinated and interlinked signaling mediated by retinoic acid-inducible gene 1 (RIG-1), RIG-1 like receptors (RLRs), Melanoma differentiation-associated protein 5 (MDA5), Toll-like receptors (TLRs), Stimulator of interferon gene (STING) *etc*. Both RIG-1 and MDA-5 were considerably studied, which were identified to detect the presence of viral nucleic acids and transduce the signal to the downstream mediators through their N-terminal Caspase recruitment domain (CARD) [68,69]. The downstream effector IFNβ promoter stimulator-1 (IPS-1, also called as the mitochondrial antiviral signaling protein MAVS) induces the canonical NF-κB signaling which leads to production of pro-inflammatory cytokines and type I IFN. Figure 2 shows the signaling molecules and the interconnected pathways in RIG-1- and MDA-5-mediated innate immune response that aid in the production of IFN and pro-inflammatory cytokines. Though additional protein interaction studies are warranted to delineate the exact molecular mechanism, the current elucidations indicate a sequence of events where the 5′ triphosphate end of RNA released from the virus first binds to the RIG-1. As a result of the induced conformational changes, the CARD domain of RIG-1 gets exposed and RIG-1 then binds to IPS-1 [70]. The precise mode of molecular interactions of MDA5 are still unclear, but the available evidence suggests that MDA5 preferentially binds to the high molecular weight double stranded RNA released from the viruses.

In Table 1, we show a brief list of molecular targets of RIG-1 and MDA5 pathway that were identified to be regulated by specific microRNAs. Many of these targets were found to either directly or indirectly affect the RIG-1/MDA5 signaling pathway, the TLR signaling or the production of interferons and regulate innate immune responses.

## 5. MicroRNAs Regulate TRIM21-Mediated Innate Immunity

The tripartite motif containing proteins (TRIMs), identified as a separate family of proteins called TRIM family of proteins, are involved in many cellular functions that regulate different signaling pathways. Specific mutations in TRIM proteins have been attributed to many human diseases. TRIM proteins characteristically contain three specific domains, the coil-coil domain, RING and B-box. Besides, they also contain either PRY and/or SPRY domains which were found to be involved in mediating protein-protein interactions. While the PRYSPRY domain of TRIM21 efficiently interacts with IgG Fc, its interaction with IgM was found to be much weaker compared to IgG. MATH and NHL domains at the C-terminal regions of TRIM proteins were also identified to mediate their interactions with other proteins.

As reviewed previously, many of the TRIM proteins have been identified to inhibit different viruses either blocking their entry or by inhibiting transcription of viral genes [71]. It is now a well-established concept that as non-enveloped viruses or bacteria enter into the human body, antibodies that originated from the adaptive immune response immediately detect these pathogens and bind to their antigenic epitopes. These antibody-bound pathogens, upon their entry into the cytosol by endocytosis, are detected by TRIM21, which is an E3 ubiquitin ligase Fc receptor. Upon binding to the pathogen bound immunoglobulins, TRIM21 then targets the pathogens to proteasomal degradation by catalyzing the formation of polyubiquitin chains. Subsequently, TRIM21 activates NF-κB, IRFs 3, 5 and 7 and AP-1 mediated innate immune signaling pathways leading to up-regulation of pro-inflammatory cytokines [72]. A schematic representation of different signaling pathways associated with innate immunity and the TRIM21 intercept as shown in Figure 2.

Sjögren’s syndrome is an autoimmune disease with wide clinical presentations ranging from chronic mild to severe symptoms. The onset of this disease is due to the expression of autoantibodies against the intracellular protein TRIM21. In an earlier study that carried out miRNA expression profiling to identify specific miRNAs as biomarkers in patients with Sjögren’s syndrome with salivary gland inflammation and dysfunction, a set of miRNAs was found to be unique for the disease [73]. In a more recent study, seven miRNAs that were predicted targets of TRIM21 were analyzed in patients with Sjögren’s syndrome which revealed up-regulation of miR-16, miR-200, miR-223 and miR-483-5p [74].

## 6. Other Epigenetic Mechanisms Induced by Invading Viruses

The entry of viral RNA and DNA inside the cell trigger a vast number of defense responses which include detection of foreign DNA or RNA, DNA damage response against the viral DNA, and epigenetic silencing of viral DNA besides induction of a plethora of intracellular proteins that aid in waging defense against the invading virus. As host cells get infected, the viral genetic elements become part of the host cell genome and depending on the transcriptional activity of the host cell, the viral genes may either start expressing the viral proteins or remain un-transcribed. Thus, in the infected cells transcriptional expression or repression of both viral and host genes are under the control of different gene regulating mechanisms, such as DNA methylation by DNMTs, post-transcriptional modifications of protein and non-coding RNAs (including microRNAs), which are collectively addressed as epigenetic modulators.

Change in the methylation status of the host cell was identified in different viral infections such as Epstein-Barr virus (EBV), Hepatitis B virus (HBV), Human papillomavirus (HPV), Kaposi’s sarcoma associated virus (KSHV) and Simian vacuolating virus (SV40) [75,76,77,78,79,80]. Subsequent to these viral infections, increased activity of DNMTs was observed leading to methylation-dependent gene silencing. The N-terminal tail region of the DNA-associated proteins “Histones”, are susceptible to different mechanisms of post-translational modifications, including methylation, acetylation, phosphorylation and ubiquitylation. Some of these modifications on histones contribute to epigenetic changes in the gene expression. Assembly of these modified histones with DNA not only inhibits DNA transcription but they also enable DNMTs-mediated DNA methylation and chromatin reorganization [81,82]. Cumulatively, these reports represent a complete picture of different epigenetic mechanisms that are involved in regulating innate immunity in the host.

## Figures and Tables

**Table 1:** A brief list of important mediators of innate immunity and their corresponding miRNAs.

Target	MicroRNA	Significance
BcL2L2	miR-29c	Promotes apoptosis
Blimp-1	miR-let-7f	Down-regulates IL-6 production
CaMKIIα	miR-148, miR-152	Promotes maturation of DCs
IKKα	miR-223, miR-15, miR-16	Activates macrophages
IKKβ	miR-199a	Inhibits TLR signaling
IKKε	miR-155	Enhances inflammation
IL-10	miR-106a	Down-regulates IL-10
IL-12	miR-155, miR-148, miR-152	Down-regulates TLR signaling
IL-12p35	miR-21	Promotes T-cell polarization
IRAK1	miR-146a	Down-regulates TLR signaling
IRAK2	miR-146a	Negatively regulates TLR signaling
IRF4	miR-125b, miR-132, miR-212	Down-regulates pro-inflammatory signaling
MyD88	miR-155	Enhances inflammation
NF-κB	miR-9, miR-218	Inhibits TLR4 mediated signaling.
Pentaxin3	miR-224	Down-regulates Ptx3 expression
PPARγ	miR-27b	Enhances LPS
PTEN	miR-21	Down-regulates PTEN, promotes IL-10 production
RIG-1	miR-545, miR-526a	Regulates RIG-1 expression
SOCS1	miR-155	Enhances inflammation
SOCS3	miR-203	Down-regulates IL-6 production
TLR2	miR-19, miR-105	Down-regulates TLR2 mediated inflammation.
TLR4	let-7e, let-7i	Down-regulates TLF4 mediated signaling
TNFα	miR-125b, miR-29c, miR-21, miR-148, miR-152	Promotes macrophage activation, multiple roles
TRAF6	miR-146a	Down-regulates TLR signaling
Tsc1 (Hamaratin)	miR-126	Targets mTOR, promotes VEGF
VLDLR	miR-23b	RIG-1 induces miR-23b production

**Table 2:** Viral miRNAs identified from miRBase.

Virus	Precursors miRNAs	Mature miRNAs
Bovine foamy virus	2	4
Bovine herpesvirus 1	10	12
Bovine herpesvirus 5	5	5
BK polyomavirus	1	2
Bovine leukemia virus	5	10
Bandicoot papillomatosis carcinomatosis virus type 1	1	1
Bandicoot papillomatosis carcinomatosis virus type 2	1	1
Duck enteritis virus	24	33
Epstein Barr virus	25	44
Herpes B virus	12	15
Human cytomegalovirus	15	26
Human herpesvirus 6B	4	8
Human immunodeficiency virus 1	3	4
Herpes Simplex Virus 1	18	27
Herpes Simplex Virus 2	18	24
Herpesvirus saimiri strain A11	3	6
Herpesvirus of turkeys	17	28
Infectious laryngotracheitis virus	7	10
JC polyomavirus	1	2
Kaposi sarcoma-associated herpesvirus	13	25
Mouse cytomegalovirus	18	29
Merkel cell polyomavirus	1	2
Mareks disease virus type 1	14	26
Mareks disease virus type 2	18	36
Mouse gammaherpesvirus 68	15	28
Pseudorabies virus	13	13
Rhesus lymphocryptovirus	36	68
Rhesus monkey rhadinovirus	7	11
Simian virus 40	1	2
Total	308	502

## Abbreviations

The following abbreviations are used in this manuscript:

CARD	caspase recruitment domain
DCs	dendritic cells
EBV	Epstein-Barr virus
HCC	hepatocellular carcinoma
IFN	interferon
IKK	IkB kinase
IKKε	inducible IkB kinase
IL	Interleukin
IPS1	IFNB promoter stimulator 1
IRAK	interleukin-1 receptor-associated kinase
IRF	interferon regulatory factor
KSHV	Kaposi’s sarcoma-associated herpesvirus
LGP2	laboratory of genetics and physiology 2
LPS	lipopolysaccharide
MAVS	mitochondrial antiviral signaling protein
MDA5	melanoma differentiation-associated protein 5
MyD88	myeloid differentiation factor 88
NLR	Nod-like receptors
PAMP	pathogen-associated molecular patterns
PRR	pathogen recognition receptors
RIG-I	retinoic acid-inducible gene 1
RLR	RIG-I-like receptors
SOCS	suppressor of cytokine signaling
STING	stimulator of interferon gene
TAK1	TGF-β-activating kinase 1
TANK	TRAF family-member-associated NF-κB activator
TBK1	TANK binding kinase 1
TLR	Toll-like receptors
TNF-α	Tumor Necrotic factor α
TRAF	TNFR-associated factor
TRAILR	tumor-necrosis factor-related apoptosis inducing ligand receptor
TRIMs	tripartite motif containing proteins
